# Processing of Functional Yoghurt-Like Product from Soymilk Supplemented by Probiotics

**DOI:** 10.1155/2022/5898537

**Published:** 2022-02-23

**Authors:** Enas Almghawesh, Samir Slik, Hussam Okkou

**Affiliations:** ^1^Food Technology Department, General Commission For Scientific Agriculture Research, Damascus, Syria; ^2^Food Science Department, Faculty of Agriculture Engineering, Damascus University, Damascus, Syria; ^3^Biodiversity Department, National Commission for Biotechnology, Ministry of Higher Education, Damascus, Syria

## Abstract

In this study, a new functional yoghurt-like product was manufactured using soymilk (sample B), mix of soymilk and cow milk (sample C), and both were compared with yoghurt of cow milk (sample A) as a control. The three yoghurt samples (A, B, and C) were processed using the traditional starter culture and *Bifidobacterium bifidum*: (A) 100% cow milk yoghurt, (B) 100% soymilk yoghurt, and (C) (50%cow milk + 50%soy milk) yoghurt. All samples were stored at 4°C for 15 days and analyzed on 1^st^, 7^th^, and 15^th^ day of the storage period. The results showed that all samples have kept a vital force of 10^6^ colony/g until the 15^th^ day of cooled storage period, and thus, achieving the feature of probiotic food. Moreover, soymilk yoghurt had the highest content of protein (3.75%) and the highest levels of unsaturated fatty acids, making it nutritious and healthy food. Furthermore, it had an acceptable taste, smell, and a firm texture. This product may be considered as a probiotic vegan and partial alternative to cow milk yoghurt. Additionally, adding probiotic bacteria prolonged the shelf-life and improved the flavor of soymilk.

## 1. Introduction

Dairy products have various nutritional and therapeutic effects on human's health. Yoghurt is one of the most consumed fermented dairy products worldwide. It has a firm coagulum, acidic taste, and many flavor compounds. It is generally produced by fermentation of animal milk using LAB (lactic acid bacteria). When the viable cell count of LAB is about (10^6^–10^8^) cfu/g, it becomes probiotic functional food [1]. Lactose intolerance and the risk of allergic reaction to animal protein, and cardiovascular diseases due to the high content of saturated fat, limit the consumption of dairy yoghurt worldwide. Moreover, the animal protein is not available in enough quantity to meet the human daily requirement of proteins in developing countries. For the reasons said above, the food industry has initiated a pilot project to develop diary yoghurt alternatives to meet the demands of vegetarians and to prevent some diseases [2].

The production of dairy yoghurt alternatives started in Hong Kong in 1940. It was limited to preparing emulsions of oily seeds and soaked legumes like soybean milk in China and extractions of coconuts and almond in Korea, Bulgaria, and Turkey. Soy milk, the most popular vegan extraction among dairy alternatives, is considered a highly nutritious and healthy food, because it has a considerable amount of high quality protein and a good level of polyunsaturated fatty acids [3]. However, marketing of soymilk is limited due to its low acceptance among consumers, because of the undesirable flavor of bean components such as methanol, hexanal, pentanal, and ethanol [4]. Flatulence caused by oligosaccharides (e.g., raffinose and stachyose) of soymilk is another limiting factor. Several studies have proved that fermentation of soymilk by lactic acid bacteria helps to remove the undesirable flavor and flatulence problems.

In addition, soymilk is considered as a typical substrate for the growth of lactic acid bacteria *Lactobacillus* together with *Bifidobacterium* by utilizing oligosaccharides in this milk and gives the special probiotic flavor to the fermented product (yoghurt). Shilpa et al. [5] reported that *Bifidobacterium* species is an essential part of colon microflora and tends to grow in anaerobic conditions in the large intestine consuming parts of food “prebiotics” which are not digested by human enzymes [6]. While species of *Lactobacillus* are aero-tolerant or anaerobic, it can inhibit the small intestine. Therefore, probiotic products containing *Lactobacilli* and *Bifidobacterium* with a viable cells count of 10^6^ (cfu/g) can affect the entire intestinal channel, providing nutritional and therapeutic effects to individuals.

The aim of this study was to develop a functional yoghurt-like product by fermentation of soymilk mixed with cow milk and supplemented with *Bifidobacterium* to compensate for the deficiency in animalistic milk and animal protein in the developing countries, especially in Syria. Therefore, it can offer a healthier and nutritious alternative to dairy products.

## 2. Materials and Methods

### 2.1. Materials

Fresh cow milk was provided by the Faculty of Agriculture farm, Damascus University. Soybeans were obtained from the Crop Department of the General Commission for Scientific Agriculture Research. Traditional starter culture of lactic acid bacteria YC-X11 (*Streptococcus thermophilus* and *Lactobacillus delbrueckii subsp bulgaricus*) was purchased from Chr. Hansen, Denmark. The starter culture of *Bifidobacterium bifidum* was purchased from Danisco, Germany.

### 2.2. Methods

#### 2.2.1. Soybean Milk Preparation

Soybeans were soaked in water for 3 hours to remove the hulls, then the soaked water was discarded, and hulls were manually removed. The dehulled beans were resoaked in 0.05% NaHco_3_ water for 20 minutes and then boiled for 5 minutes at 100°C (NaHco_3_ was used to blanch soybeans). The boiling water was discarded, and the beans were rinsed with pure water. Water was added to beans by a ratio of 6 : 1, respectively. The bean with water was mixed at high speed for 10 minutes, then, the slurry was filtered through double layers of cheese cloths. The pure extraction was heated at 85°C for 30 minutes [7].

#### 2.2.2. Enrichment of Starter Culture

Enrichment of starter culture was done because of the small size of manufactured yoghurt samples. Two closed bottles containing 100 ml of zero-fat milk supplemented with 0.2% yeast extract, and 0.1% sucrose, were used to activate the starter cultures. Samples were heated to 90°C for 10 min and then cooled to 42°C. One sample was inoculated with 1 g of starter culture (*Lactobacillus delbrueckii subsp bulgaricus* and *Streptococcus thermophilus*), and the other sample was inoculated with 1 g of *Bifidobacterium bifidum*. Both bottles were then incubated at 45°C to reach the pH point 4.6 (2.5 hours for YC-X11 and 2 hours for *Bifidobacterium bifidum*) then stored at 4°C to be used later in processing yoghurt samples [8].

#### 2.2.3. Manufacturing of Yoghurt Samples

The following three samples of yoghurt were prepared:

A: Yoghurt made from 100% cow milk (1000 ml)

B: Yoghurt made from 100% soymilk(1000 ml)

C: Yoghurt made from (50%*cow* *milk* + 50%*soymilk*) (1000 ml)

Milk samples were heated to 85°C for 30 min, cooled rapidly to 43°C, inoculated with enriched cultures (3% of raw milk: 1.5% from enriched YC-X11, 1.5% from enriched *Bifidobacterium bifidum*), and mixed well manually after closing the bottles. The bottles were incubated at 43°C to get pH point 4.6 after 2.5 hours and then stored at 4°C for 15 days. The initial vital force for the enriched cultures was at the level of (10^6^ cfu/g).

## 3. Methods of Analyses

### 3.1. Microbiological Analyses

Each yoghurt sample was tested for the vital count of each bacterial species, and the microbiological analyses were carried out in triplicate at three time points: day 1 (time 0), 7, and 15 of cooled storage for (*Streptococcus thermophilus*, *Lactobacillus delbrueckii subsp bulgaricus*, and *Bifidobacterium bifidum*) using petri dishes. Pepton water was used to prepare serial dilutions starting from 10^−1^ to 10^−9^, and 1 ml of each dilution placed in the petri dish, then 15 ml of the suitable media for each bacterial species was added to the petri dish. Acidified MRS Agar medium (pH = 5.2) [9] was used for enumeration of *Lactobacillus delbrueckii subsp bulgaricus,* incubated anaerobically at 44°C for 72 h. M17 Agar medium was used for *Streptococcus thermophilus* incubated at 37°C for 72 h, and BSM Agar supplemented with BSM supplement SIGMA-ALDRICH was used for enumeration of *Bifidobacterium bifidum* incubated anaerobically at 37°C for 5 days. The colonies of each species were counted in each sample to obtain the total vital bacterial count (total vital force).

### 3.2. Chemical Analyses

The chemical analyses were performed in triplicate at day 1 (time 0), 7, and 15 of cooled storage. The total protein, fat contents, and acidity of yoghurt samples were estimated according to the standard methods [10].

Total protein was determined according to Sorensen method using titration with formaldehyde by calibrating 10 ml of yoghurt + 0.4 ml potassium oxalate + 1 ml phenolphthalein indicator with 0.1 N NaOH and formaldehyde. Total fat was determined using Gerber's method by adding 10 ml sulfuric acid + 11 ml yoghurt + 1 ml amyl alcohol in butyrometers, respectively. Then, the butyrometers were agitated and inverted to mix the three liquids before centrifugation for 4-5 min. The meniscus was adjusted to obtain the reading of the fat ratio. Acidity was determined by measuring lactic acid contents by titrating a mix of 10 ml sample and 10 ml distilled water with NaOH 0.1 N using phenolphthalein as an indicator, until the light pink color remained for two minutes.

According to [11], the quality and quantity of fatty acids were determined in yoghurt samples on the day 15 of storage period using gas chromatography with flame ionization detector (FID), and the carrier gas was a mix of nitrogen and helium obtained from PEAK Manufacturers. Rose-Gottlieb method was used to extract the lipids. All samples were treated with ammonia to dissolve the protein and with ethyl alcohol to precipitate the proteins. Fat was extracted using diethyl ether and petroleum ether, then, ethers were evaporated. Fatty acids were esterified by potassium methylate. The temperature (260°C), pressure (1 bar), and column temperature (80°C) were applied. The fatty acids in samples were identified by comparison with those obtained from SIGMA-ALDRICH standard and SASMO 3770/2019 (Syrian Arab Organization for Standardization and Metrology).

### 3.3. Sensory Evaluation

Sensory evaluation of the yoghurt samples was done according to Likert scale: 1: strongly agree, 2: agree, 3: neutral, 4: disagree, 5: strongly disagree, for the following criteria: color, smell, taste, texture, and general acceptability [12]. Ten entraining candidates were invited to evaluate the samples in triplicate on the day 1 (time 0), 7, and 15 of cooled storage.

### 3.4. Statistical Analyses

All statistical analyses were performed in *GenStat®* 12 using factorial design analyses and two-way ANOVA to test the significance level with (LSD *p* ≤ 0.05).

## 4. Results and Discussion

### 4.1. Microbiological Analyses of Yoghurt Samples

Microbiological analyses were presented as a decimal logarithm of colony forming units per gram (log cfu/g) for the bacterial count in every sample.

The counts of all three bacterial species used in the manufactured yoghurt showed that all yoghurt samples kept a vital force of bacterial count at a level of 6 log (cfu/g) at least from the beginning of storage period day 1 (time 0) till the end of storage period (Table 1). There was a significant difference (*p* < 0.05) between means of bacterial counts for the three samples A, B, and C. The initial vital force for the three enriched cultures was at the level of (10^6^ cfu/g), then, it differed according to the type of fermented milk and the activity of cultures within it. Comparing with cow yoghurt (A), sample (C) showed a good type of probiotics product with a bacterial count of (8.41 ± 0.1) log (cfu/g), and sample (B) also gained a significant vital force of total bacterial count close to probiotics (6.86 ± 0.02) log (cfu/g). This indicates that *Bifidobacterium* and lactic acid bacteria (*Lactobacillus delbrueckii subsp bulgaricus* and *Streotococcus thermophilus*) could cohabitate in a fermented vegan substrate like soymilk and work cooperatively to produce a probiotic product, as well as maintaining an adequate number of living microorganisms when consumed. This was consistent with the definition of probiotics products [13]. *Bifidobacterium* secretes *α*-galactosidase that breaks down oligosaccharides of soymilk thus reducing flatulence caused by these oligosaccharides. Previous researches reported production of functional yoghurt from soymilk using strains of *Lactobacillus* and *Bifidobacterium* [14, 15].

The bacterial count of *Streptococcus thermophilus* was presented as a decimal logarithm of colony forming units per gram (log cfu/g) in every sample, Table 2. There was a significant difference (*p* < 0.05) between means of *Streptococcus thermophilus* counts for three samples A, B, and C that was attributable to the difference in the ability of *Streptococcus thermophilus* to ferment different types of milk. All yoghurt samples maintained a vital force of bacterial count at a level of at least 6 log (cfu/g) along all storage period from day 1 (time 0) till the end of storage period (15 days). This is due to the ability of *Streptococcus thermophilus* to utilize all kinds of sugars found in the milk used, including soymilk.

The bacterial count of *Lactobacillus delbrueckii subsp bulgaricus* was presented as a decimal logarithm of colony forming units per gram (log cfu/g) in every sample. As shown in Table 3, all yoghurt samples kept the vital force of *Lactobacillus delbrueckii subsp bulgaricus* count; at least a level of 6 log (cfu/g) throughout storage period (i.e.,15 days). There was a significant difference (*p* < 0.05) for *Lactobacillus delbrueckii subsp bulgaricus* counts among three samples A, B, and C at day 1 (time 0). The highest mean of bacterial count was in sample A, followed by sample B and sample C. Similar results were previously reported [15].

The bacterial count of *Bifidobacterium bifidum* was presented as a decimal logarithm of colony forming units per gram (log cfu/g) in every sample. All yoghurt samples exceeded the threshold for probiotic product count of *Bifidobacterium bifidum* 6 log (cfu/g) during the storage period (Table 4). There was a significant difference (*p* < 0.05) in *Bifidobacterium bifidum* counts for the three samples A, B, and C that could be explained by the ability of this bacterial species to ferment all types of milk used to prepare samples. Sample A has the highest count of *Bifidobacterium bifidum*, followed by sample B and sample C.

### 4.2. Chemical Analyses of Yoghurt Samples

The results of the chemical analyses of yoghurt samples showed an increase in the mean values of protein, fat, and acidity during storage period due to the partial loss of moisture in all manufactured yoghurt samples (Table 5). Similar findings were reported by [16].

Sample B (100% soymilk yoghurt) contained significantly more protein (3.75% ± 0.01, *p* < 0.05) compared to samples C (50%cow milk + 50%soy milk) and sample A (100% cow milk yoghurt). This suggests that soymilk yoghurt may substitute cow milk yoghurt in terms of meeting daily protein requirements for people who are strict vegan [17].

Although fermentation time was the same for all samples (2.5 hours), the differences in acidity were significant among the samples along the storage period. This was due to postacidification phenomenon at low temperatures 4 ± 1°C [18] and variation in activity of starter culture within yoghurt samples during the storage period. The highest value of acidity was in sample (A) (1.16% ± 0.01), and the lowest was in sample (B) (0.63% ± 0.01). *Bifidobacterium bifidum* has the ability to ferment soymilk oligosaccharide to the same extent as fermenting cow milk lactose, the preferred substrate [19]. For fat content, soymilk (sample B) had the lowest value (2.94 ± 0.01), while cow milk yoghurt (sample A) had the highest value (3.63% ± 0.01), which was mainly due to high fat percentage in cow milk and this agreed with the results stated by [20].

### 4.3. Fatty Acid Composition in Yoghurt Samples

The three yoghurt samples were analyzed using gas chromatography (GC) to determine the qualitative and quantitative content of fatty acids. All analyses were performed at the end of the storage period.

Table 6 showed that soymilk yoghurt (sample B) had the highest values of all unsaturated fatty acids except palmitoleic acid, and the lowest values of all saturated fatty acids followed by the mixed yoghurt (sample C). Furthermore, soymilk yoghurt contained the highest level of the essential fatty acids; oleic acid (26.80% ± 0.1), linoleic acid (32.03% ± 0.1), and *α*- linolenic acid (3.74% ± 0.1), followed by the mixed yoghurt. Hence, adding soymilk may help lowering the ratio of saturated fatty acids to unsaturated fatty acids thus lowering the cholesterol level, specifically low-density cholesterol (LDL) in yoghurt, yielding healthier yoghurt [21, 22].

### 4.4. Sensory Analysis of Yoghurt Samples

Sensory evaluation of yoghurt samples was performed in triplicate on the days (1, 7, and 15) of the storage period as shown in Table 7. The sensory test showed that there was a significant difference among the samples according to all sensory criteria, color, smell, taste, texture, and overall acceptability regardless of the storage period. Acidity level had a large impact on test, while protein content had significant impact on texture and coagulum. In addition, natural variations related to consumer preferences contributed to these results.

Sample (A) had the highest scores for taste (4.70 ± 0.4), and for smell (4.20 ± 0.7), followed by sample (C) with a score (4.10 ± 0.3) for both taste and smell, throughout the storage periods. Although reduced the undesirable flavors caused by n-hexanal and pentanal, fermenting bacteria (i.e., lactic acid bacteria and *Bifidobacterium*) did not yield in a desirable soymilk yoghurt taste, which received the lowest score (2.80 ± 0.4) among the three samples [23]. For color, there was a significant difference among samples during storage period. Sample (A) had the highest score (4.90 ± 0.3) with the white color. Sample (B) had the lowest score with the yellowish creamy color that mainly due to lecithin; a brown-yellowish phospholipid [24]. For texture, sample (B) had the highest score (5.00 ± 0) followed by sample (C) (4.90 ± 0.3). It was noted that when the proportion of soymilk increased in the manufactured yoghurt, the consistency of the yogurt increased, and the separation of yoghurt whey decreased. This is because of globulin, the main soymilk protein that can entrap water inside the matrix more strongly than casein in cow milk. The molecules of casein contain large spaces between each other due to their large size causing further separating of the whey [25].

## 5. Conclusion

Soymilk yoghurt is a good nondairy yoghurt-like product. It has functional and healthy effects as the vital force of the final product survived at the level of probiotic functional product count (10^6^ cfu/g) which extends the shelf- life of the yoghurt. Additionally, soymilk yoghurt had acceptable results in sensory properties (taste and smell), because probiotic bacteria masked the bean flavor of soymilk in the final product. On the other hand, soymilk yoghurt had the highest content of protein and unsaturated fatty acids making it a healthier food product. It had a firmer texture but had an undesirable taste that can be modified using natural flavors and coloring agents to get a functional, tasty, and healthy product.

## Figures and Tables

**Table 1:** The total count of starter bacteria in yoghurt samples during storage at 4 ± 1°C.

Samples	Storage period (day)		
	1	7	15
A (100% cow milk yoghurt)	9.43^a^ ± 0.02	7.57^c^ ± 0.05	7.22^d^ ± 0.04
B (100% soymilk yoghurt)	6.86^e^ ± 0.02	6.72^f^ ± 0.06	6.57^g^ ± 0.02
C (50%cow milk + 50%soy milk) yoghurt	8.41^b^ ± 0.1	7.23^d^ ± 0.06	6.54^g^ ± 0.01

Different small letters in the same row refer to a significant difference at (*p* < 0.05). Different small letters in the same column refer to a significant difference at (*p* < 0.05). Values explained means ± SD for all samples.

**Table 2:** The total count of *Streptococcus thermophilus* in yoghurt samples during storage at 4 ± 1°C.

Samples	Storage period (day)		
	1	7	15
A (100% cow milk yoghurt)	6.36^a^ ± 0.02	6.24^b^ ± 0.05	6.20^c^ ± 0.04
B (100% soymilk yoghurt)	6.21^c^ ± 0.02	6.15^de^ ± 0.06	6.06^f^ ± 0.02
C (50%cow milk + 50%soy milk) yoghurt	6.19^c^ ± 0.1	6.16^d^ ± 0.06	6.13^e^ ± 0.01

Different small letters in the same row refer to a significant difference at (*p* < 0.05). Different small letters in the same column refer to a significant difference at (*p* < 0.05). Values explained means ± SD for all samples.

**Table 3:** Total count of *Lactobacillus delbrueckii subsp bulgaricus* in yoghurt samples during storage.

Samples	Storage period (day)		
	1	7	15
A (100% cow milk yoghurt)	6.15^a^ ± 0.02	6.05^d^ ± 0.05	6.05^d^ ± 0.04
B (100% soymilk yoghurt)	6.09^bc^ ± 0.02	6.07^cd^ ± 0.06	6.04^d^ ± 0.02
C (50%cow milk + 50%soy milk) yoghurt	6.11^b^ ± 0.1	6.05^d^ ± 0.06	6.04^d^ ± 0.01

Different small letters in the same row refer to a significant difference at (*p* < 0.05). Different small letters in the same column refer to a significant difference at (*p* < 0.05). Values explained means ± SD for all samples.

**Table 4:** The total count of *Bifidobacterium bifidum* in yoghurt samples during storage at 4 ± 1°C.

Samples	Storage period (day)		
	1	7	15
A (100% cow milk yoghurt)	9.35^a^ ± 0.02	7.16^d^ ± 0.05	7.13^d^ ± 0.04
A (100% cow milk yoghurt)	6.59^e^ ± 0.02	6.41^f^ ± 0.06	6.06^g^ ± 0.02
C (50%cow milk + 50%soy milk) yoghurt	7.57^b^ ± 0.1	7.39^c^ ± 0.06	6.43^f^ ± 0.01

Different small letters in the same row refer to a significant difference at (*p* < 0.05). Different small letters in the same column refer to a significant difference at (*p* < 0.05). Values explained means ± SD for all samples.

**Table 5:** Some chemical properties of the yoghurt samples (mean ± SD).

Samples	Storage period (day)	Protein%	Fat%	Acidity%
A (100% cow milk yoghurt)	1	3.14^g^ ± 0.01	3.58^c^ ± 0.01	0.75^c^ ± 0.01
	7	3.16^f^ ± 0.01	3.60^b^ ± 0.01	1.09^b^ ± 0.01
	15	3.20^e^ ± 0.01	3.63^a^ ± 0.01	1.16^a^ ± 0.01
B (100% soymilk yoghurt)	1	3.72^b^ ± 0.01	2.89^h^ ± 0.01	0.51^g^ ± 0.02
	7	3.73^b^ ± 0.01	2.91^g^ ± 0.01	0.57^f^ ± 0.01
	15	3.75^a^ ± 0.01	2.94^f^ ± 0.01	0.63^e^ ± 0.01
C (50%cow milk + 50%soy milk) yoghurt	1	3.44^d^ ± 0.02	3.23^e^ ± 0.01	0.65^de^ ± 0.02
	7	3.45^d^ ± 0.02	3.25^e^ ± 0.01	0.67^d^ ± 0.02
	15	3.47^c^ ± 0.02	3.28^d^ ± 0.01	0.73^c^ ± 0.01

Different small letters in the same column refer to a significant difference at (*p* < 0.05).

**Table 6:** Fatty acid composition of yoghurt samples (mean ± SD values).

Saturated fatty acids	Mean values of saturated fatty acid in yoghurt samples%		
	A (100% cow milk yoghurt)	B (100% soymilk yoghurt)	C (50%cow milk + 50%soy milk) yoghurt
Butyric C4:0	4.23^a^ ± 0.1	n.d	3.74^b^ ± 0.1
Caproic C6:0	2.17^a^ ± 0.1	n.d	1.33^b^ ± 0.1
CaprylicC8:0	2.30^a^ ± 0.1	n.d	1.43^b^ ± 0.1
Capric C10:0	3.63^a^ ± 0.1	n.d	2.13^b^ ± 0.1
Lauric C12:0	4.70^a^ ± 0.1	n.d	2.83^b^ ± 0.1
MyristicC14:0	10.60^a^ ± 0.1	4.20^c^ ± 0.1	9.83^b^ ± 0.1
PalmiticC16:0	42.40^a^ ± 0.1	22.67^c^ ± 0.1	28.77^b^ ± 0.1
Stearic C18:0	4.72^a^ ± 0.3	3.76^c^ ± 0.1	4.30^b^ ± 0.1
Unsaturated fatty acids	Mean values of unsaturated fatty acid in yoghurt samples%		
	A	B	C
Myristoleic	0.52^b^ ± 0.4	1.73^a^ ± 0.1	0.85^b^ ± 0.1
Palmitoleic	2.76^a^ ± 0.1	0.24^c^ ± 0.2	1.07^b^ ± 0.01
Oleic *ω*9	19.67^c^ ± 0.5	26.80^a^ ± 0.1	22.67^b^ ± 0.5
Linoleic *ω*6	2.13^c^ ± 0.1	32.03^a^ ± 0.1	14.10^b^ ± 0.1
*α*- Linolenic *ω*3	n.d	3.74^a^ ± 0.1	1.23^b^ ± 0.1

Different small letters in the same row refer to a significant difference at (*p* < 0.05). Different small letters in the same column refer to a significant difference at (*p* < 0.05). n.d: not detected. Each sample was statistically analyzed for each type of fatty acid using one-way ANOVA test followed by post hoc L.S.D to see the significant differences (*p* < 0.05).

**Table 7:** Sensory evaluation of yoghurt samples during storage period.

Storage period (days)	Treatments	Quality attribute				
		Color	Smell	Taste	Texture and body	Overall acceptability
1	A	4.90^a^ ± 0.3	4.20^a^ ± 0.7	4.70^a^ ± 0.4	3.60^b^ ± 0.8	4.40^a^ ± 0.6
	B	2.20^c^ ± 0.4	3.60^b^ ± 0.4	2.80^c^ ± 0.4	5.00^a^ ± 0	2.97^c^ ± 0.2
	C	3.80^b^ ± 0.4	4.10^ab^ ± 0.3	4.10^b^ ± 0.3	4.90^a^ ± 0.3	4.00^b^ ± 0.3
7	A	4.80^a^ ± 0.4	3.70^a^ ± 0.6	4.20^a^ ± 0.4	3.60^b^ ± 0.8	4.08^a^ ± 0.3
	B	1.90^c^ ± 0.8	3.00^b^ ± 0.4	2.70^b^ ± 0.6	5.00^a^ ± 0	3.15^b^ ± 0.2
	C	3.00^b^ ± 0.8	3.50^ab^ ± 0.5	4.10^a^ ± 0.5	4.90^a^ ± 0.3	3.87^a^ ± 0.3
15	A	4.60^a^ ± 0.5	3.70^a^ ± 0.6	4.10^a^ ± 0.3	3.60^b^ ± 0.8	4.03^a^ ± 0.3
	B	1.90^c^ ± 0.8	3.00^b^ ± 0.5	2.70^b^ ± 0.6	5.00^a^ ± 0	3.10^b^ ± 0.2
	C	3.00^b^ ± 0.8	3.50^ab^ ± 0.5	4.00^a^ ± 0.4	4.90^a^ ± 0.3	3.85^a^ ± 0.3

Different small letters in the same column refer to a significant difference at (*p* < 0.05).

## Data Availability

The data set is available from the corresponding author upon request.
